# The urban energy balance of a lightweight low-rise neighborhood in Andacollo, Chile

**DOI:** 10.1007/s00704-016-1922-7

**Published:** 2016-10-07

**Authors:** Ben Crawford, E. Scott Krayenhoff, Paul Cordy

**Affiliations:** 1 Department of Geography, University of British Columbia, Vancouver, Canada; 2 Present Address: Department of Meteorology, University of Reading, Reading, UK; 3 Present Address: School of Geographical Sciences and Urban Planning, Arizona State University, Tempe, AZ USA; 4 Department of Mining Engineering, University of British Columbia, Vancouver, Canada; 5 Present Address: Cordy Geoscience, Squamish, BC Canada

## Abstract

Worldwide, the majority of rapidly growing neighborhoods are found in the Global South. They often exhibit different building construction and development patterns than the Global North, and urban climate research in many such neighborhoods has to date been sparse. This study presents local-scale observations of net radiation (Q_*_) and sensible heat flux (*Q*_*H*_) from a lightweight low-rise neighborhood in the desert climate of Andacollo, Chile, and compares observations with results from a process-based urban energy-balance model (TUF3D) and a local-scale empirical model (LUMPS) for a 14-day period in autumn 2009. This is a unique neighborhood-climate combination in the urban energy-balance literature, and results show good agreement between observations and models for Q_*_ and *Q*_*H*_. The unmeasured latent heat flux (*Q*_*E*_) is modeled with an updated version of TUF3D and two versions of LUMPS (a forward and inverse application). Both LUMPS implementations predict slightly higher *Q*_*E*_ than TUF3D, which may indicate a bias in LUMPS parameters towards mid-latitude, non-desert climates. Overall, the energy balance is dominated by sensible and storage heat fluxes with mean daytime Bowen ratios of 2.57 (observed *Q*_*H*_/LUMPS *Q*_*E*_)–3.46 (TUF3D). Storage heat flux (*ΔQ*_*S*_) is modeled with TUF3D, the empirical objective hysteresis model (OHM), and the inverse LUMPS implementation. Agreement between models is generally good; the OHM-predicted diurnal cycle deviates somewhat relative to the other two models, likely because OHM coefficients are not specified for the roof and wall construction materials found in this neighborhood. New facet-scale and local-scale OHM coefficients are developed based on modeled *ΔQ*_*S*_ and observed Q_*_. Coefficients in the empirical models OHM and LUMPS are derived from observations in primarily non-desert climates in European/North American neighborhoods and must be updated as measurements in lightweight low-rise (and other) neighborhoods in various climates become available.

## Introduction

The rapid pace of urban development globally has been well documented, and the case for process-based studies of the urban energy balance has been made extensively in the urban climate literature. There have also been several calls for increased study of developing tropical and sub-tropical urban areas because these cities have been underrepresented in urban climate research and their urban populations are forecast to grow at over three times the rate of mid- and high-latitude cities (e.g., Roth 2007). Within these (sub-) tropical developing cities, the population living in informal, unplanned neighborhoods made of lightweight construction materials (thin, un-insulated walls and roofs) and often with minimal formal services (e.g., water, electricity, transportation) is currently growing 10 % per year globally (UN-HABITAT 2008). At present, over one billion people worldwide are estimated to live in these neighborhoods (UN-HABITAT 2008) and it is important to incorporate these areas into environmental models across a range of scales (micro-global) for a variety of applications (e.g., urban planning, resource consumption, thermal comfort, air quality, hydrology).

According to the local climate zone classification scheme (Stewart and Oke 2012), these neighborhoods can be classified as “lightweight low-rise” (LL). Although this classification scheme was originally devised with canopy-layer urban-heat island studies in mind, it summarizes important neighborhood characteristics and provides a useful descriptive framework for energy-balance research.

The urban energy balance can be expressed for a neighborhood-scale volume (including the 3-day urban surface and air volume extending from the surface through the roughness sub-layer) as (Oke 1988):

1 $$ {Q}_{\ast }+{Q}_F={Q}_H+{Q}_E+\varDelta {Q}_S+\varDelta {Q}_A $$

where the net radiation (Q_*_) and anthropogenic heat flux (*Q*_*F*_) are energy inputs to the system, which are partitioned between sensible heat flux (*Q*_*H*_), latent heat flux (*Q*_*E*_), and storage heat flux (*ΔQ*_*S*_). *ΔQ*_*A*_ is energy flux from advection and is typically assumed to be zero based on assumptions of a continuous, extensive, and homogeneous study surface, although in areas with larger (mesoscale) circulations, this assumption is unlikely to hold (e.g., Pigeon et al. 2007).

The terms of the energy balance of lightweight low-rise neighborhoods are expected to contrast with other local climate zones due to differences in construction materials, whose reduced heat storage capacity will affect *ΔQ*_*S*_; differences in residential energy systems, fuel type, and usage patterns, which will influence *Q*_*F*_; and different tropical and sub-tropical climate regimes, which will modify partitioning between *Q*_*H*_ and *Q*_*E*_ as well as input from *Q*_*F*_.

Although LL neighborhoods are home to one in three urban residents worldwide, a survey of urban energy-balance studies reveals a lack of observational data from these neighborhoods. Although there have been a handful of energy-balance measurements from urban areas in sub-tropical and tropical climates (see Roth (2007) for a review), the majority of studies have been representative of more established neighborhoods in developed North American cities. Only a measurement site in Ouagadougou, Burkina Faso (Offerle et al. 2005), is considered to be a LL neighborhood.

In Ouagadougou, eddy-covariance measurements from a residential neighborhood show the *Q*_*H*_ and *ΔQ*_*S*_ terms are dominant through the day as there is little surface moisture available for *Q*_*E*_ in the semi-arid Sahel climate during dry season. The mean midday Bowen ratio (*β = Q*_*H*_ */Q*_*E*_) was observed to be 3.7, and a bottom-up method to model *ΔQ*_*S*_ based on building surface temperatures shows an early peak in *ΔQ*_*S*_ (~1000 LST) due to the high thermal conductivity and low heat capacity of the buildings.

In terms of modeling, local-scale numerical model development and evaluation efforts have also concentrated on mid-latitude cities and neighborhoods to date (Masson et al. 2002; Lemonsu et al. 2004; Oleson et al. 2008; Kawai et al. 2009). Models have been developed for and verified with data from these mid-latitude cities, and there have not been observations to test existing model performance in different types of neighborhoods under different climate regimes.

The first objective of this study is to address this gap in observations and present partial energy-balance measurements (Q_*_ and *Q*_*H*_) from a LL neighborhood in an arid climate in Andacollo, Chile. This is a unique neighborhood-climate configuration in the urban energy-balance literature. The second objective is to model energy-balance terms *Q*_*H*_, *Q*_*E*_, and *ΔQ*_*S*_ with a combination of empirical models (OHM (Grimmond and Oke 1999) and LUMPS (Grimmond and Oke 2002)). Thirdly, we compare observations and OHM/LUMPS results to a process-based urban energy-balance model (TUF3D; Krayenhoff and Voogt 2007), whose input parameters should be less scenario-dependent than those of OHM/LUMPS. Similarly, Offerle et al. (2005) found that another process-based urban climate model with similar physics (town energy balance; Masson 2000) compared well with measurements in a dry lightweight low-rise neighborhood in Ouagadougou, Burkina Faso. The OHM, LUMPS, and TUF3D models were originally developed for mid-latitude cities and have been verified with mid-latitude datasets and have not been tested with this type of neighborhood in this type of climate.

## Methods

### Setting

Andacollo is located in the Elqui Provice, Coquimbo Region of northern Chile (30° 13′ 55.66″ S, 71° 04′ 42.38″ W), approximately 50 km east of the Pacific Ocean in the foothills of the Andes mountains. The town is situated at an elevation of approximately 1050 m in a basin surrounded by terrain with elevations of 1200–1400 m. The regional climate is classified as sub-tropical desert (*BWk*) according to the Köppen classification system. Annual precipitation in the region is 108 mm and primarily falls during the winter months (May–August) while average annual temperature is 14.5 °C (high 17.5 °C in January, low 12 °C in June).

According to the most recent available census count from 2002, the town has a population of approximately 10,000 and covers an area of 310 km^2^ (mean population density is 32 people km^2^). The local economy is based on gold and copper mining, and there are a variety of extraction activities ranging from independent subsistence miners using hand tools to large-scale industrial mines owned by multinational corporations. This measurement campaign was part of a study to measure and model dispersion of mercury vapor resulting from indigenous mining practices (Cordy et al. 2013).

**Fig. 1:**
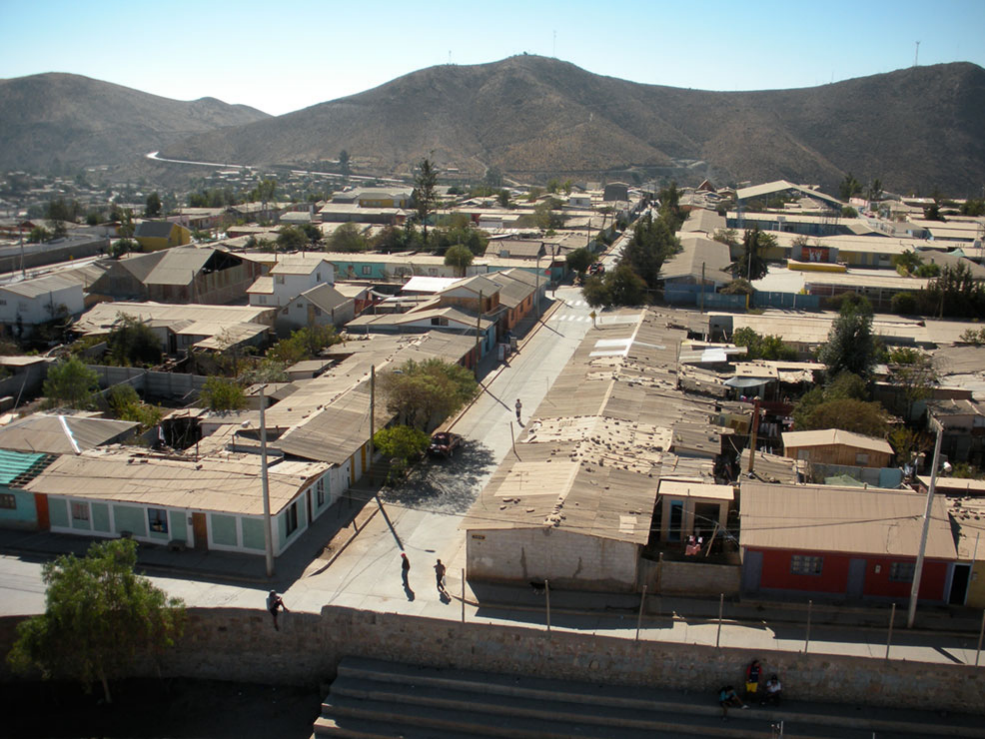
Photo of Andacollo study area during April 2009 from approximately 20 m a.g.l. View is towards the north. The location of the photo is shown in Fig. 2

**Fig. 2:**
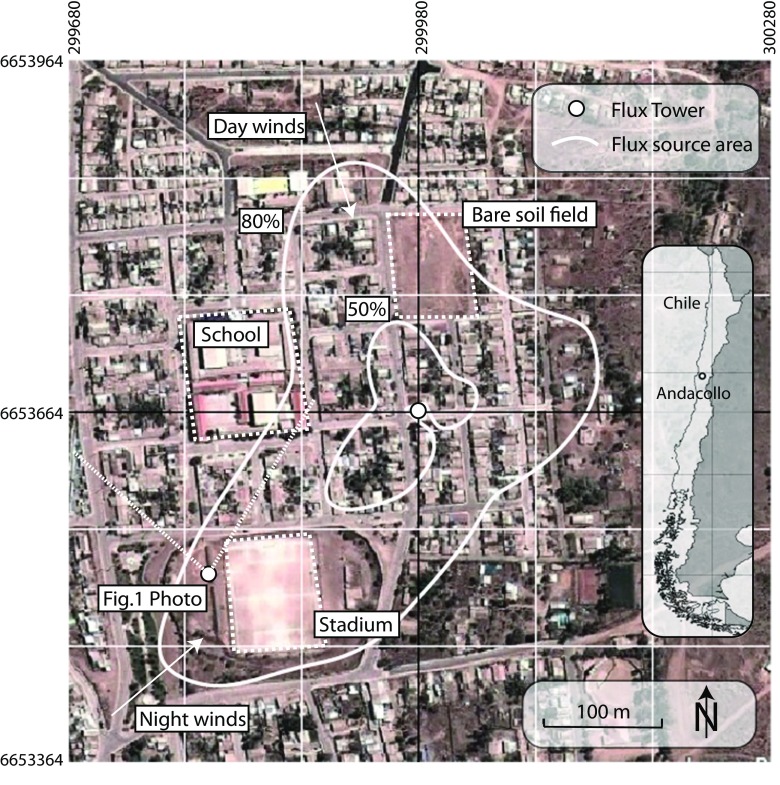
Aerial photo of Andacollo study area with cumulative flux source area weighting overlay. Cumulative weighted contour lines are shown at 50 and 80 % intervals. The tower is at the center of the image at the intersection of the *vertical and horizontal black lines*. UTM coordinates (Zone 19 J) and a 100 m × 100 m grid are overlaid on the study area. Day and nighttime mean wind directions (not scaled for velocity) are labeled; see Fig. 2 for hourly wind vectors (velocity and direction). Background image is from October 8, 2011 (Google Earth)

The study neighborhood is located in a residential area of town (Figs. 1 and 2). Mean building height (*h*) is 4 m and buildings are primarily constructed from concrete blocks with corrugated metal roofs and are organized into blocks (mean block dimensions 60 × 80 m). In between blocks, streets are paved with light-colored concrete and are patterned in a grid generally aligned N-S and E-W (mean street width = 8 m). Along the street fronts, there is often no space in between buildings; though in the block interiors, there are small yards with bare soil surface and vegetation (average height ≈4 m, with individual trees up to 6 m). Building interiors are generally heated by portable electric radiators or wood fireplaces, but these are assumed to be largely inactive during the study period because of relatively mild air temperatures (>8 °C). Motor vehicle traffic in the area is very light.

The neighborhood is not an unplanned, informal settlement. Services such as water and electricity are provided, garbage and sewage are removed, and the neighborhood is organized into a regular grid pattern with paved, impervious roads. In these respects, this neighborhood is not representative of many informal LL settlements. In terms of built materials and thermal properties, though, especially the thin metal roofs and concrete block walls, measurements from this site provide a representative LL dataset for testing urban climate models.

### Eddy-covariance and air temperature measurements

Local-scale observations of the study neighborhood were measured from a custom-built mast at a height (*z*) of 12 m (*z/h* = 3). Three-dimensional wind velocities and sonic virtual temperature were recorded at 10 Hz from an ultrasonic anemometer (81000, R.M. Young Company, Traverse City, MI, USA) and net radiation (NR-Lite, Kipp & Zonen, Delft, The Netherlands), air temperature, and relative humidity (HMP 45 A, Vaisala, Finland) were sampled at 1-s intervals and recorded as 5-min averages.

Sensible heat flux (*Q*_*H*_) was calculated using the eddy-covariance method:

2 $$ {Q}_H={c}_p\rho \overline{w\hbox{'}t\hbox{'}}, $$

where *c*_*p*_ is the specific heat capacity of air (J kg^−1^ K^−1^), *ρ* is air density (kg m^−3^), and $\overline{w\hbox{'}t\hbox{'}}$ is the mean covariance of vertical wind (*w’*) and air temperature (*t’*) fluctuations. The 10 Hz data were block-averaged in 30-min intervals and 2-d coordinate rotation was performed using in-house processing software (Crawford and Christen 2014) and a correction was applied to account for the path-length of the sonic anemometer (Moore 1986).

Indoor and outdoor air temperatures were also measured using stand-alone temperature loggers (HOBO, Onset Corp., Bourne, MA, USA). The indoor temperature sensor was mounted on a wall shelf at 1.5 m height in the central living area of a representative home in the flux source area. The outdoor sensor was shielded, passively ventilated, and mounted at 1.5 m height at the base of the flux tower. Both sensors sampled air temperatures at 1-s intervals and recorded 5-min averages.

**Fig. 3:**
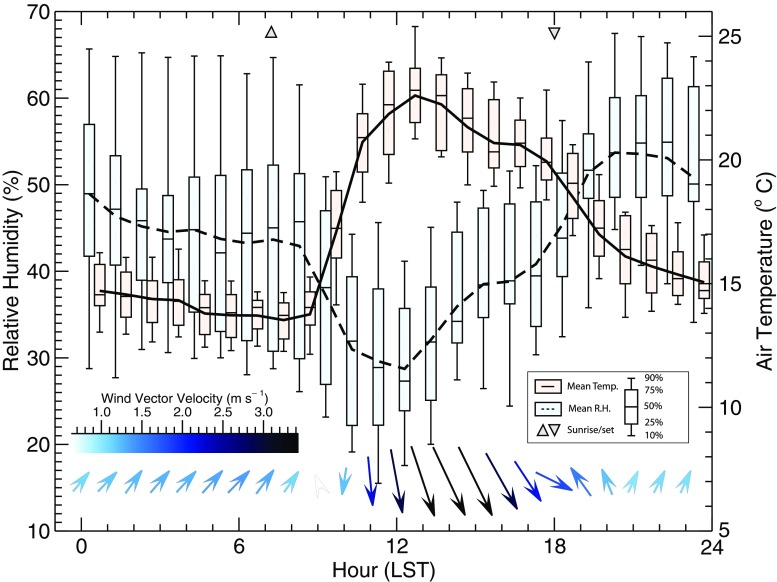
Observed hourly mean air temperature, relative humidity, and wind vectors from the Andacollo flux tower (12 m agl), April 21–May 4, 2009

Data presented here were measured from April 21–May 4, 2009, and during this period, the mean air temperature recorded on the tower (12 m) was 17.0 °C (daily high mean = 22.6 °C, daily low mean = 13.5 °C), relative humidity ranged from 20 to 60 %, there was no precipitation, and the day-to-day weather was stable and consistent. Winds were generally calm with daytime flows from the NW (mean daytime velocity = 3.0 m s^−1^) and nighttime flow from SW (mean nighttime velocity = 1.0 m s^−1^) (Fig. 3). The 24-h mean of hourly ensemble mean measured indoor temperatures during the study period was 19.5 °C, with a maximum of 24.4 °C at 1600 h and minimum of 14.9 °C at 0700 h.

### Flux source-area land cover analysis

Plan-area land cover classification of the turbulent flux source area is used to characterize the surface for energy-balance modeling (Table 1). Land cover classification was performed manually from a georeferenced aerial photograph (image date October 8, 2011, GoogleEarth). All geospatial analysis in this study was performed using freely available images and open-source GIS software (Quantum GIS).

**Table 1:** Turbulent flux source area surface characteristics determined from spatial analysis and source area modeling (Section 2.3) and used as input into LUMPS and TUF3D models

	Source area-weighted mean (m^2^ m^−2^)	Mean height (m a.g.l.)
Building plan-area landcover (*λ*_*B*_)	0.49	4
Vegetation plan-area landcover (*λ*_*V*_)	0.15	4
Bare soil plan-area landcover (*λ*_*S*_)	0.13	0
Impervious plan-area landcover (*λ*_*I*_)	0.23	0
Canyon aspect ratio (*h/w*)	0.5	–

The digital aerial photograph is formatted with separate red, green, and blue layers that are combined for full-color visualization according to the RGB color-coding system. For each RGB layer, individual image pixels have a value ranging from 0 (darkest) to 255 (lightest). A binary vegetation raster (1 = vegetated, 0 = non-vegetation) classification was performed using the green band of the aerial photograph. In Andacollo, vegetation stands out as much darker than light-colored roofs, streets, and soil, particularly on the green band. Using a visual comparison with the full-color image, a range of green band pixel values was selected to represent vegetation and individual pixels with values that fall within this range are classified as vegetation. Given the predominance of light-colored surfaces in this area (streets, building roofs, bare soil), green pixel values occupy a discreet range for vegetation, and there is minimal overlap with other surfaces. This classification was manually checked against the full-color aerial photograph to ensure minimal attribution error. Additionally, a sensitivity analysis was conducted to determine how errors in vegetation land cover classification affect the modeled fluxes (Section 3.1).

Next, a layer of polygons representing building plan area was manually drawn over the aerial photograph using GIS software. Most blocks have several buildings side-by-side without space between them, and these groupings are considered as a single polygon. There are also several unpaved areas within the study neighborhood, including a park, a football stadium playing field, and a school with an open courtyard playground. The surface in these areas is light-colored bare soil. Polygons were manually drawn over these areas using GIS software.

Building and soil polygon layers are then rasterized as binary layers at the same resolution as the vegetation layer. If after this step, an individual cell is classified as both vegetation and building or soil, the cell is classified as vegetation. This assumes tree canopies overhang buildings and soil areas. Finally, impervious concrete roads are assumed to cover all remaining unclassified surface pixels.

Turbulent flux source areas for *Q*_*H*_ measurements were then modeled at 30-min time steps using the source area model by Kormann and Meixner (2001). As input, the model uses 30-min mean wind direction (measured from the tower with the sonic anemometer), surface roughness length (determined from surface morphometry), lateral dispersion (standard deviation of the crosswind velocity measured by the sonic anemometer), and atmospheric stability (Obukhov length, *L*, determined from sonic anemometer measurements). The output of the model is an individual source area for each 30-min flux measurement that is a surface weighted by the likelihood of its contribution to the measured flux at the tower. On average, the modeled source area 50 % level lies within 100 m of the tower and 86 % lies within the land cover analysis domain (Fig. 2). Portions of the source area falling outside this domain are weighted according to the neighborhood average of each surface land cover. Individual source areas are overlaid on the land cover dataset to determine the weighted land cover composition of each 30-min source area (e.g., Schmid and Lloyd 1999; Christen et al. 2011). This is then used to determine the mean source area-weighted land cover composition of the eddy-covariance measurements (Table 1).

### Modeling *ΔQ*_*S*_ with OHM

Typically, energy-balance studies using the eddy-covariance method also include fast-response measurements of water vapor concentration to observe *Q*_*E*_. Observationally, the storage term is then considered to be the residual net radiation (if *Q*_*F*_ is neglected) after *Q*_*H*_ and *Q*_*E*_ are accounted for (*ΔQ*_*S*_ = Q_*_ − *Q*_*H*_ − *Q*_*E*_), with all measurement errors included in *ΔQ*_*S*_ (i.e., the residual). In this study, no measurements of *Q*_*E*_ are available to use the residual method and instead *ΔQ*_*S*_ is explicitly modeled using several methods.

First, the storage heat flux, *ΔQ*_*S*_, is modeled using the objective hysteresis model (OHM) (Grimmond and Oke 1999). OHM has been tested and used extensively in a range of urban environments has been established as a robust method to model *ΔQ*_*S*_ (e.g., Roberts et al. 2006).

OHM is based on observed hysteresis patterns between Q_*_ and *ΔQ*_*S*_ (determined as the residual of Q_*_, *Q*_*H*_, and *Q*_*E*_ measurements) for seven cities with varying morphometric surface properties and climate conditions (Grimmond and Oke 1999). The model calculates *ΔQ*_*S*_ as the sum of storage contributions of *n* individual surface types (*i*) at the surface:

3 $$ \varDelta {Q}_S={\displaystyle \sum_{i=1}^n{\lambda}_i\left[{a}_{1i}{Q}_{*}+{a}_{2i}\frac{\delta {Q}_{*}}{\delta t}+{a}_{3i}\right]}. $$

The model coefficients *a*_*1*_, *a*_*2*_, and *a*_*3*_ are found in published data for common surface types (Table 2). For this study, six surface types are used and weighted according to their fraction of total surface area: mixed forest, dry bare soil, impervious concrete, dry asphalt shingle roof, N-S canyon, and E-W canyon. There is uncertainty introduced to the model from choice of coefficients for each surface. In particular, the roof coefficients are a source of uncertainty in this study because we could not source coefficients in the literature that are specific to the corrugated metal roofs found in this study area. The dry asphalt shingle roof type was selected as an analog because coefficients are derived from direct measurements (instead of modeled) taken over residential roofs (instead of commercial or industrial) during dry conditions (Meyn and Oke 2009).

**Table 2:** OHM coefficients for individual surfaces to calculate ∆*Q*_*S*_. Individual surfaces are scaled by surface fractional coverage to a representative local-scale average (Section 2.4)

Surface	*a*_*1*_	*a*_*2*_	*a*_*3*_	Source
Mixed forest	0.11	0.11	−12.3	McCaughey (1985)
Dry bare soil	0.35	0.43	−36.5	Fuchs and Hadas (1972)
Dry asphalt shingle rooftop	0.12	0.25	−5.0	Meyn and Oke (2009)
Impervious concrete	0.85	0.32	−28.5	Asaeda and Ca (1993)
N-S canyon	0.32	0.01	−27.7	Nunez (1974)
E-W canyon	0.71	0.04	−39.7	Yoshida et al. (1991)
Lightweight, dry, un-insulated corrugated metal roof (TUF3D)	0.22	0.52	−16.7	This study
Lightweight, un-insulated concrete walls (TUF3D)	0.18	0.51	−10.5	This study
Lightweight low-rise neighborhood (TUF3D)	0.40	0.35	−58.2	This study
Lightweight low-rise neighborhood (L2)	0.42	0.15	−44.7	This study

### Modeling *Q*_*H*_, *Q*_*E*_, and *ΔQ*_*S*_ with LUMPS

Two different implementations of the local-scale urban meteorological parameterization scheme (LUMPS; Grimmond and Oke 2002) are used to model *Q*_*H*_, *Q*_*E*_, and *ΔQ*_*S.*_ This model is selected because it is widely used, is relatively straightforward to implement, and has been established as a robust method to calculate turbulent fluxes in a range of urban areas. In the first version (L1), the model is run in its typical forward implementation to calculate *Q*_*H*_ and *Q*_*E*_ using measured Q_*_ and *ΔQ*_*S*_ provided by OHM (Section 2.4). In the second implementation (L2), the model is inverted to model *ΔQ*_*S*_ and *Q*_*E*_ using measured Q_*_ and *Q*_*H*_ as input.

#### Forward application L1 to model *Q*_*H*_ and *Q*_*E*_

In this version of LUMPS, observed Q_*_ and *ΔQ*_*S*_ modeled by OHM are used as inputs to calculate *Q*_*H*_:

4 $$ {Q}_H=\frac{\left(1-\alpha \right)+\left(\gamma /s\right)}{1+\left(\gamma /s\right)}\left(Q*-\varDelta {Q}_S\right)-b $$

and *Q*_*E*_:

5 $$ {Q}_E=\frac{\alpha }{1+\left(\gamma /s\right)}\left(Q*-\varDelta {Q}_S\right)+b, $$

where γ is the psychometric constant, *s* is the slope of the saturation vapor pressure-temperature curve (determined from measured air temperature), and *b* and *α* are empirical parameters specified for use in urban environments (Grimmond and Oke 2002).

These calculations are dependent on the choice of *b* and *α* parameters, particularly *α*. Grimmond and Oke (2002) back-calculate values of *b* and *α* for a range of urban sites and recommend a constant value of 3 W m^−2^ be used for *b*. The *α* value depends on the surface moisture conditions and linear regression relations between the *α* value and *λ*_*V*_ are given by Grimmond and Oke (2002) based on several other urban sites in the literature. For this study, *α* values were calculated using these linear regressions for each 30-min flux-averaging period based on *λ*_*V*_, with a mean *α* for all periods of 0.29.

Another caveat is that the anthropogenic heat flux *Q*_*F*_ is assumed to be primarily measured in *Q*_*H*_ and so is not considered explicitly to avoid double counting. The magnitude of *Q*_*F*_ is expected to be small in this area due to low population density, relatively mild temperatures (i.e., low interior space-heating demand), and light vehicle traffic. The *Q*_*F*_ term is explicitly considered in the TUF3D model (Section 2.6).

#### Inverted application L2 to model *ΔQ*_*S*_ and *Q*_*E*_

This implementation takes advantage of the observed Q_*_ and *Q*_*H*_ in this study to rearrange Eq. 4 to solve for *ΔQ*_*S*_ *.* This *ΔQ*_*S*_ is then used to solve for *Q*_*E*_ using Eq. 5. This implementation of LUMPS in a sense is a residual approach with the measured energy-balance residual of Q_*_ and *Q*_*H*_ (and measurement errors) divided between *Q*_*E*_ and *ΔQ*_*S*_.

### TUF3D

A version of the temperatures of facets in 3-D (TUF3D) urban energy-balance model (Krayenhoff and Voogt 2007) optimized for regular building arrays was used to independently model the volume energy fluxes and indoor and outdoor air temperatures for the duration of the measurement period. TUF3D fluxes and (sub-) facet surface temperatures have been evaluated against measurements from two mid-latitude cities, and the model has subsequently been applied to study the impacts of ground cover/surfacing on building energy performance (Yaghoobian et al. 2010, Yaghoobian and Kleissl 2012), evaluate radiation models (Krayenhoff et al. 2014), and provide surface temperatures for remote sensing research (Krayenhoff and Voogt 2016). The purpose of the current modeling exercise is twofold—to evaluate the ability of TUF3D to represent the measured fluxes (Q* and *Q*_*H*_) and temperatures of a novel urban land cover-climatic zone combination and to use it to estimate the relative magnitudes of the unobserved fluxes (principally, Δ*Q*_*S*_ and *Q*_*E*_).

#### Model description and development

TUF3D was designed as a dry, three-dimensional microscale urban energy-balance model with a focus on radiative exchange. Plane parallel facets (roofs, walls, streets/canyon floor) are split into identical square patches, each of which exchanges shortwave and longwave radiation, sensible heat, and conduction heat. Incident solar radiation on each patch is solved via ray tracing, and diffuse receipt and reflections are computed using view factors and matrix inversion. Profiles of wind speed and air temperature are calculated as a function of urban morphology and above-canyon forcing, and these quantities drive sensible heat exchange from patches at each height. Patches are divided into layers parallel to the surface, each with unique thermal properties, and heat conduction between the exterior and interior (or deep) surfaces is computed. Further details about the model and its evaluation are available in Krayenhoff and Voogt (2007).

TUF3D has since been optimized for regular arrays and refined in several ways. Yaghoobian et al. (2010) included a simple representation of the latent heat effects of low vegetation, and Yaghoobian and Kleissl (2012) added the ASHRAE toolkit and other features in order to include the building internal energy balance (Pedersen et al. 2001). Here, the original model (Krayenhoff and Voogt 2007) is used with the following developments and refinements:

i. Internal building energy balance

Diurnal variation of indoor air temperature (*T*_*INT*_) is significant in Andacollo. Furthermore, the internal building energy balance is simpler than typical North American structures in that energy-intensive heating and cooling are not common. Hence, the evolution of *T*_*INT*_ is modified to reflect these realities:

6 $$ {T}_{INT}^{m+1}={T}_{INT}^m+\frac{\varDelta t}{c_{INT}}\cdot \left[{Q}_{G, roof}^{m+1}+{Q}_{G, wall}^{m+1}+{Q}_{F,INT}+{Q}_{Floor}^{m+1}\right]+\varDelta {T}_{V,INT}^{m+1}, $$

where *m* is the time step number, Δ*t* is the time step, *c*_*INT*_ is the areal heat capacity of the internal air (W m^−2^ K), *Q*_*G* , *roof*_ and *Q*_*G* , *wall*_ are the exchange at the inner layers of the roofs and walls (Krayenhoff and Voogt 2007), *Q*_*F,INT*_ is a prescribed indoor anthropogenic heat flux, Δ*T*_*V,INT*_ is an ventilation term (see below) and *Q*_*Floor*_ is the exchange with the building floor and interior possessions:

7 $$ {Q}_{Floor}^{m+1}=\frac{2}{\varOmega_{INT}}\cdot \left({T}_{INT}^m-{T}_{Floor}^m\right), $$

where Ω_*INT*_ is the resistance to convective and radiative transfer (Masson et al. 2002) also used for ${Q}_G^{roof}$ and ${Q}_G^{wall}$, set here to 0.123 m^2^ K W^−1^, and the factor of 2 is an estimate of the enhanced contact between the indoor elements and the indoor air. The floor temperature is computed as follows:

8 $$ {T}_{Floor}^{m+1}={T}_{Floor}^m-\frac{\varDelta t}{c_{Floor}}\cdot {Q}_{Floor}^{m+1}, $$

where *c*_*Floor*_ is assumed to be 150 % (to account for household possessions) of the heat capacity of the thermally active layer of the building floor, defined here as the depth at which the diurnal temperature variation is reduced to 1/e of that at the surface (Stull 1988):

9 $$ {c}_{Floor}=1.5\cdot C\cdot \sqrt{\frac{86400\cdot k}{\pi \cdot C}}, $$

where *k* and *C* are the thermal conductivity and volumetric heat capacity of the material, respectively.

i. Ventilation

Ventilation is included in the formulations of internal and external air temperature because the buildings in Andacollo typically lack air tightness. The fraction of indoor air exchanged at each time step is computed as follows:

10 $$ \varDelta {v}^{m+1}=\frac{\varDelta t}{3600}\cdot a\cdot {U}_{can}^m, $$

where *U*_*can*_ is the (mid-) canyon wind speed, and *a* is a coefficient, chosen to be 1.0 s m^−1^ here (one air exchange per hour per m s^−1^ of canyon wind speed). Internal and external air temperature changes are then as follows:

11 $$ \varDelta {T}_{V,INT}^{m+1}=\varDelta {v}^{m+1}\cdot \left({T}_{can}^m-{T}_{INT}^m\right), $$

12 $$ \varDelta {T}_{V, can}^{m+1}=-\varDelta {v}^{m+1}\cdot \frac{\lambda_P}{1-{\lambda}_P}\cdot \left({T}_{can}^m-{T}_{INT}^m\right), $$

where $\varDelta {T}_{V, can}^{m+1}$ is added to Eq. 11 in Krayenhoff and Voogt (2007) in analogous fashion to Eq. 6 above.

ii. Simple treatment of evaporating surfaces (vegetation)

Andacollo is vegetated primarily with trees and other vegetation with vertical structure, at road edges or in courtyards. The evaporative effects of vegetation were included here in a very simple manner based on Yaghoobian et al. (2010). That is, the latent heat flux density for select canyon patches (wall or road) was simply given as:

13 $$ {Q}_E=\frac{1}{\beta}\cdot {Q}_H, $$

where *Q*_*H*_ is computed as in Krayenhoff and Voogt (2007; Eq. 14), and *β* is the Bowen ratio, chosen here to be 0.1 (borderline “oasis” due to the dry conditions, assuming mostly trees with access to subsurface water). Vegetation plan-area coverage is ≈15 % in Andacollo, which corresponds to ≈25 % of the ground surface area. Hence, wall and canyon floor patches are chosen at random such that 25 % of the patches include this *Q*_*E*_ term in their energy balance. Both wall and canyon patches are important to include as vegetation shades and replaces them both as the active surface during the daytime. Latent heat flux is set to zero for all patches during nighttime (defined as *K*↓ < 1 W m^−2^). Furthermore, the surface thermal conductivity of these “vegetated” patches was set to 0.1 W m^−1^ K^−1^ to appropriately shift energy exchange to the turbulent and radiative fluxes at the expense of conduction.

#### Simulation development

Forcing data was measured where possible and modeled otherwise. Air temperature and humidity and wind speed and direction were all measured at 12 m. Measured pressure varied only slightly and was assumed constant throughout at 896 hPa. Incoming longwave was modeled from measured temperature and humidity following Prata (1996), and direct and diffuse shortwave was modeled with a scheme based on the Bird and Hulstrom (1981) model and reported in Iqbal (1983), assuming clear skies (which prevailed over the period). Both radiation schemes demonstrate excellent performance across a range of datasets (Prata 1996; Gueymard and Myers 2008).

Land cover parameters were extracted from Google Earth (Section 2.3): building plan-area fraction (*λ*_*B*_) was 0.49, street height-to-width ratio (*H*/*W*) was ≈0.5 but was increased to 0.67 to account for the courtyards (whose H/W ≈ 1.0), and street directions are rotated 5° counterclockwise from the cardinal directions. Roughness length, displacement height, and frontal area index are not specified and are therefore computed automatically as in Krayenhoff and Voogt (2007). Roof and road roughness lengths are 0.05 m, indicating roughness on these surfaces is of the order of 0.5 m, and the ratio of the roughness lengths for momentum and heat is chosen to be 50, similar to the ratio found for bluff roughness of this scale (Kanda et al. 2007). Wall roughness as defined in Krayenhoff and Voogt (2007) is set to 1.0 (concrete).

Thermal and radiative parameters were drawn from two sources, principally. The “local climate zone” parameter ranges for the “lightweight low-rise” zone (Stewart et al. 2014) were used as initial guidance. The authors’ personal experience and photos were then used to refine the parameter choices (Tables 2 and 3) based on tabulated values in Oke (1987). Roofs are primarily corrugated, aged metal (presumed steel, and underlain by some wood and/or concrete). Hence, emissivity (thermal conductivity) values were chosen to be substantially higher (lower) than for new, polished, flat metal (Table 4). Walls are relatively thin and composed of medium density concrete with some windows, and ground level is about 50 % concrete over dry soil (roads) and 50 % exposed sandy soil (courtyards, etc.). Radiative and thermal parameters are necessarily rough spatial averages—given the uncertainty in material parameters and their coverages, a more refined weighting technique (e.g., Salamanca et al. 2009) was not employed.

**Table 3:** Radiative parameters of the urban surface facets used in TUF3D. Sources: Oke 1987; Stewart and Oke 2012

	Roof	Floor (street)	Wall
Albedo	0.18	0.30	0.30
Emissivity	0.80	0.90	0.90

An indoor anthropogenic heat flux (*Q*_*F,INT*_) of 5 W m^−2^ is added to the building space during nighttime (*K*↓ < 1 W m^−2^), and inhabitants will tend to be in their homes. This represents a heat flux about 1.5 times larger than the metabolic output of the city’s population divided by the area of residential floor space and is additionally intended to include pets, cooking, electricity use, and any other anthropogenic sources.

The simulation was run for the entire period (April 21–May 4, 2009), and the ensemble mean values were compared to their measured counterparts, excluding the first day (April 21) to permit for model spin-up.

## Results and discussion

### Q_*_ and *Q*_*H*_

**Fig. 4:**
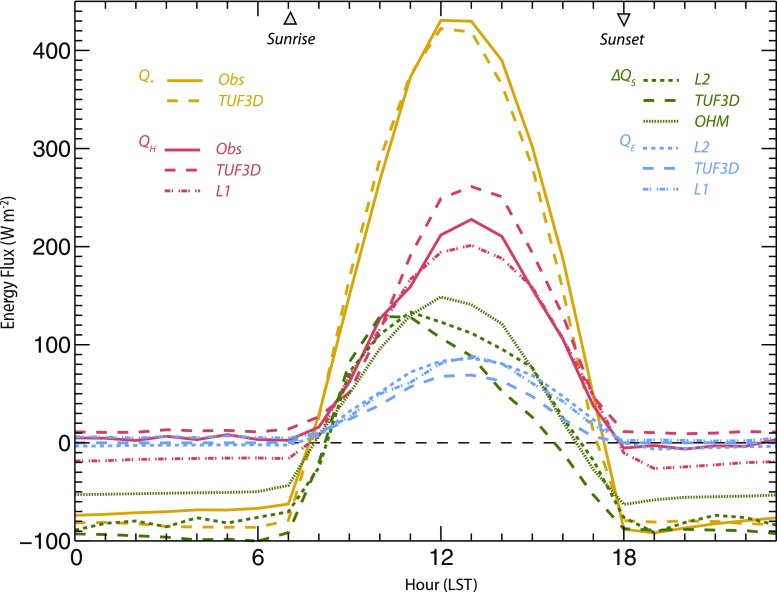
Hourly mean observed and modeled components of the surface energy balance. Observed Q_*_ and *Q*_*H*_ are ensemble means of measurements from individual hours during April 21–May 4, 2009. Error bars are omitted for clarity. See text for details of TUF3D, OHM, L1, and L2 model settings

Observations of Q_*_ conform to an expected diurnal pattern based on solar radiation input and clear skies (Fig. 4). From 0000 to 0700 LST, mean Q* is −70.6 W m^−2^. After sunrise (~0700 LST), Q* increases, becomes positive after 0800 and reaches a maximum at 1300 LST (430.1 W m^−2^). After 1300 LST, Q* decreases throughout the afternoon, becomes negative at 1730 LST, and is on average −82.4 W m^−2^ for the remainder of the 24-cycle.

Observed *Q*_*H*_ also follows an expected diurnal cycle. Magnitudes of *Q*_*H*_ are small and slightly positive from 0000 to 0600 h (mean = 5.5 W m^−2^) then begin to increase after sunrise. Peak *Q*_*H*_ occurs at 1300 (233.7 W m^−2^) and values decline through the afternoon. After sunset (~1800 h), observed *Q*_*H*_ magnitudes are small and slightly negative (mean = −6.2 W m^−2^), indicating a cool surface and likely stable atmospheric conditions. This is unsurprising, given the general lack of thermal mass in this neighborhood to maintain a heat source from storage release.

This diurnal pattern of *Q*_*H*_ is similar to that observed in a residential area of Ouagadougou, Burkina Faso (Offerle et al. 2005). At this site, mean *Q*_*H*_/Q_*_ from 1100 to 1400 was 0.41, compared to 0.49 during the same hours in Andacollo. In Ouagadougou, however, *Q*_*H*_ remained positive for an hour after sunset, offsetting large storage heat release (−*ΔQ*_*S*_).

**Fig. 5:**
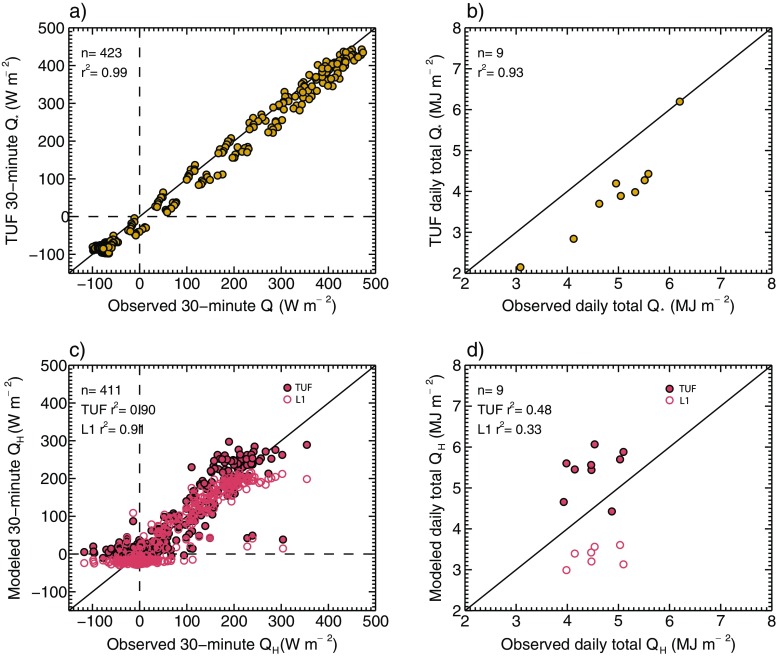
Comparison of observed and TUF3D-modeled 30-min Q_*_ (**a**) and *Q*_*H*_ (**c**) (W m^−2^) and daily totals of Q_*_ (**b**) and *Q*_*H*_ (**d**) (MJ m^−2^ day^−1^) for the 9-day study period. Statistical model summary is also given in Table 5

The TUF3D model qualitatively performs well in reproducing the diurnal pattern and magnitudes of observed hourly Q_*_ and *Q*_*H*_ (Fig. 4). Agreement is particularly good with respect to Q_*_ (daytime mean difference 11.2 W m^−2^), although the model slightly underestimates Q_*_ at night, by 13.5 W m^−2^. Observed daily totals of Q* range from 3.1–6.3 MJ m^−2^ (Fig. 5).

For *Q*_*H*_, TUF3D is successful in replicating the observed diurnal pattern, though the model overestimates observations by 26.7 W m^−2^ on average in the afternoon and early evening (1100–1900 LST). The moderate, monodirectional daytime (afternoon) winds are suggestive of a larger (local-regional) scale circulation, which may in part generate the afternoon overestimation of *Q*_*H*_ due to removal of heat by advection below measurement height (Masson et al. 2002; Pigeon et al. 2007).

LUMPS-modeled *Q*_*H*_ (L1) also shows good agreement with observations, especially during daytime. From 1200 to 1500 LST, modeled *Q*_*H*_ underestimates observations by −14.9 W m^−2^ on average. This implementation is dependent on *ΔQ*_*S*_ as input and is sensitive to uncertainties in the OHM model (Section 3.2). Overnight (0000–0500 LST), the L1 model underestimates observations by −21.6 W m^−2^ on average. This nocturnal difference could in part be due to anthropogenic heat flux measured as *Q*_*H*_ by the eddy-covariance system, though LUMPS also inherently includes influence of *Q*_*F*_ in modeled *Q*_*H*_ because it is based on observations. Though the absolute nighttime difference is not great, the difference in sign could be critical for some applications (e.g., air quality). Negative *Q*_*H*_ suggests stable conditions and lack of vertical mixing, whereas positive *Q*_*H*_ is representative of a slightly unstable atmosphere and vertical exchange.

Observed-model differences are also likely related to the dynamic turbulent flux source areas. This site was subject to consistent wind flow patterns with overnight and morning winds from the southwest and daytime winds from the northwest (Figs. 2 and 3). Any differences in land cover and resident behavior between flux source areas will be reflected in the eddy-covariance measurements, while the models are more representative of an unchanging local-scale average.

Quantitatively, comparisons between modeled and observed Q_*_ and *Q*_*H*_ are characterized by low-magnitude mean bias error (*MBE*) and high coefficient of determination (*r* ^*2*^) values, respectively (Table 5). Root-mean-square error (RMSE) appears somewhat less flattering, especially for *Q*_*H*_; however, these values are similar to average RMSE magnitude across 30+ urban surface models reported in a model inter-comparison by Grimmond et al. (2011). Notable, especially for *Q*_*H*_, is the small systematic (*RMSEs*) and large unsystematic (*RMSEu*) RMSE. According to Willmott (1981), this suggests that overall RMSE cannot be reduced without substantial changes to the model (e.g., its resolution in time or space). In the case of *Q*_*H*_, the time series is replete with variation at the averaging time scale (30 min) which is likely related to phenomena not captured by TUF3D, LUMPS, or any urban canopy models for that matter (e.g., nocturnal *Q*_*H*_ intermittency related to the stable surface-layer conditions). Conversely, LUMPS has significant *RMSEs* in addition to its *RMSEu*, suggesting that there is opportunity for model improvement or better parameter specification. Additionally, sensitivity simulations indicate TUF3D Q_*_ and *Q*_*H*_ are not overly sensitive to variations in vegetation landcover fraction. With an increase of *λ*_*V*_ of 5 %, median hourly difference in modeled Q_*_ is 2.5 W m^−2^ (0.8 %) and for *Q*_*H*_ median, difference is 6.6 W m^−2^ (3.5 %) during midday (1000–1500 h).

**Table 4:** Thermal properties of the urban surface facets used in TUF3D. Layer 1 borders the outdoor atmosphere. Sources: Oke 1987; Stewart and Oke 2012

	Heat capacity (C) (×10^6^ J m^−3^ K^-1)^	Conductivity (k) (W m^−1^ K^−1^)	Thickness (m)
Roof			
Layer 1	3.50	5.00	0.005
Layer 2	1.30	0.60	0.005
Layer 3	1.30	0.60	0.010
Layer 4	1.30	0.60	0.010
Floor (street)			
Layer 1	1.30	0.45	0.010
Layer 2	1.30	0.45	0.025
Layer 3	1.30	0.40	0.065
Layer 4	1.30	0.30	0.150
Wall			
Layer 1	1.00	0.60	0.010
Layer 2	1.00	0.60	0.020
Layer 3	1.00	0.60	0.030
Layer 4	1.00	0.60	0.040

### *ΔQ*_*S*_ and *Q*_*E*_

**Fig. 6:**
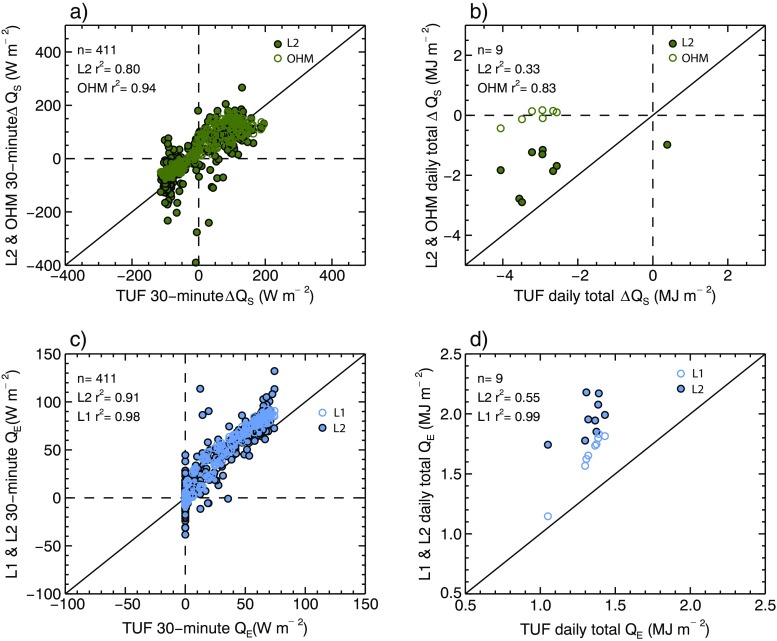
Comparison of L1-, L2-, and TUF3D-modeled 30-min Δ*Q*_*S*_ (**a**) and *Q*_*E*_ (**c**) (W m^−2^) and daily totals of Δ*Q*_*S*_ (**b**) and *Q*_*E*_ (**d**) (MJ m^−2^ day^−1^) for the 9-day study period. Statistical model summary is also given in Table 5

There is in general very good agreement between models with respect to *ΔQ*_*S*_ *.* All three models show negative overnight *ΔQ*_*S*_ values (storage energy release to the atmosphere) followed by increases after sunset that lag Q_*_. Both TUF3D and L2 have peak values at 10- 1100 (140 W m^−2^), before a decline through the afternoon (Fig. 4). This diurnal pattern is explained by the low heat capacities and high thermal conductivities of building construction materials, and it demonstrates similar magnitude and hysteresis to measurements for a similar neighborhood in Ouagadougou, Burkina Faso (Offerle et al. 2005). The thin, un-insulated concrete walls (and metal roofs, to an extent) quickly accumulate energy after sunrise and rapidly release the stored heat after sunset. The overall agreement between the two methods is very good, though TUF3D predicts slightly less *ΔQ*_*S*_ than L2 in the early morning and afternoon (0500–0700 and 1200–1800), which is also reflected in lower daily total *ΔQ*_*S*_ values (Fig. 6). Both L2 and TUF3D models also predict negative daily totals of *ΔQ*_*S*_, suggesting that the system is losing energy. This is to be expected during southern hemisphere autumn.

**Table 5:** Summary statistics of model performance in comparison to observed Q_*_ and *Q*_*H*_ during the 9-day comparison period. Statistics are from 30-min mean values (*n* = 432). RMSE is root-mean-square error and *RMSEs* (*RMSEu*) is systematic (unsystematic) RMSE. MBE is mean bias error, and *d* is the index of agreement (Willmott 1981)

	Q* (W m^−2^)	*Q*_*H*_ (TUF3D) (W m^−2^)	*Q*_*H*_ (LUMPS L1) (W m^−2^)
RMSE	20.3	44.4	39.4
RMSEs	6.7	1.4	20.6
RMSEu	19.1	44.4	33.6
MBE	3.7	1.3	−15.6
*r* ^*2*^	0.99	0.80	0.83
*d*	1.00	0.94	0.95

In contrast to TUF3D and L2, OHM predicts a peak in *ΔQ*_*S*_ at 1200 LST, 1 h later than L2 and TUF3D. This likely is due to use of model coefficients representative of different building materials than what are found in the study area. In particular, the dry asphalt shingle roof coefficients used in the model are representative of material with greater heat capacity than thin, metal sheeting found in the study area.

New facet-scale hysteresis coefficients for OHM are calculated for roofs and walls in this neighborhood based on facet-scale *ΔQ*_*S*_ results from TUF3D (Table 2). New local-scale OHM coefficients for the entire neighborhood based on L2 and TUF3D *ΔQ*_*S*_ and observed Q_*_ are also calculated. Coefficients (*a*_*1*_, *a*_*2*_, *a*_*3*_) were fit to Eq. 3, where *n* = 1, *λ*_*i*_=1, Q_*_ is measured at the tower, and *ΔQ*_*S*_ is either the TUF3D model output per m^−2^ of facet (roof or wall, respectively), or the local-scale *ΔQ*_*S*_ model output from L2 or TUF3D. The equation was fit to ensemble hourly means during the study period (*n* = 24) and $\frac{\delta Q*}{\delta t}$ is estimated as follows:

$\frac{\delta Q*}{\delta t}=\left[Q{*}_{t+1}-Q{*}_{t-1}\right]*0.5$. There is some uncertainty with this approach at facet-scale because the measured Q_*_ is representative of other surfaces (i.e., local-scale neighborhood) besides the facet surface of interest. Also, this analysis does not control for wind speed, which is shown to affect storage and OHM coefficients (Grimmond and Oke 1999; Meyn and Oke 2009).

TUF3D, L1, and L2 models produce similar diurnal patterns with respect to *Q*_*E*_, though the latent heat flux is overall a relatively minor component of the energy balance due to a lack of surface water, precipitation, and vegetation cover (Fig. 4). All three models predict minimal overnight *Q*_*E*_ and a daytime peak at 1300 LST (TUF3D = 70 W m^−2^, L1 = 90 W m^−2^, L2 = 86 W m^−2^). Observations in Ouagadougou follow a similar diurnal pattern but *Q*_*E*_ magnitude is reduced, perhaps because vegetation cover is about 30 % less than in the present flux source area (Offerle et al. 2005). During the afternoon (1300–1800 LST), both L1 and L2 applications of LUMPS allocate more energy to *Q*_*E*_ compared to TUF3D (by 13.0 W m^−2^ on average). TUF3D does not include anthropogenic latent heat, which is implicitly included in both LUMPS-derived *Q*_*E*_ calculations.

**Fig. 7:**
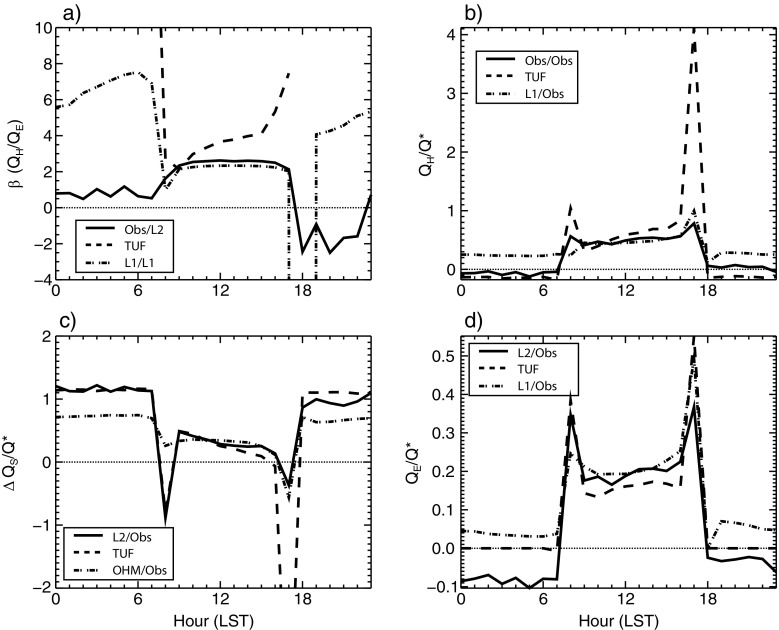
Observed and modeled energy-balance flux ratios **a** β (*Q*_*H*_/*Q*_*E*_), **b** *Q*_*H*_/Q*, **c** *ΔQ*_*S*_ /Q*, and **d** *Q*_*E*_ /Q*. Ratios are calculated from ensemble mean hourly values during the study period. Note the different *y*-axis ranges between plots

The differences in energy allocation between *Q*_*E*_ and *Q*_*H*_ result in slightly different Bowen ratios (*β*) (Fig. 7, Table 6). Mean mid-daytime (1000–1500 LST) *β* values for observed and L2-modeled fluxes is 2.56, for L1-modeled fluxes is 2.42, and for TUF3D-modeled fluxes is 3.46. This indicates TUF3D considers the Andacollo surface to be drier than LUMPS (both L1 and L2). This is likely partially due to the manner in which the models represent surface moisture. In LUMPS calculations, surface moisture is represented by the *α* parameter. According to Grimmond and Oke (2002), there is a linear relation between surface moisture (approximated by *λ*_*V*_) and *α.* Higher *α* values are associated with greater *λ*_*V*_ and presumably more surface moisture.

**Table 6:** Mean midday values of energy-balance flux ratios. Values are from 1000 to 1500. Ratios in columns Obs, L2 and Obs, L1, OHM are combinations of modeled and observed (Obs) values. Obs describes observed *Q*_*H*_ and Q* values (Section 2.2), L1 refers to the forward implementation of LUMPS (Section 2.5.1), L2 is the inverse LUMPS (Section 2.5.2), and OHM is the objective hysteresis model (Section 2.4). TUF3D settings are described in Section 2.6. Also, see Fig. 7 for 24-h time series of ratios

Ratio	Obs, L2	Obs, L1, OHM	TUF3D
*Q*_*H*_ */Q*_*E*_ *(β)*	2.57 (Obs/L2)	2.45 (L1/L1)	3.46
*Q*_*H*_ /Q_*_	0.49 (Obs/Obs)	0.47 (L1/Obs)	0.55
*ΔQ*_*S*_ /Q_*_	0.27 (L2/Obs)	0.32 (OHM/Obs)	0.29
*Q*_*E*_ /Q_*_	0.19 (L2/Obs)	0.20 (L1/Obs)	0.15

TUF3D-modeled *Q*_*E*_ is moderately sensitive to variations in vegetation landcover fraction. With an increase of *λ*_*V*_ of 5 %, median hourly difference in modeled *Q*_*E*_ during midday (1000–1500 h) is 11.4 W m^−2^ (17 %). Sensitivity to the least certain parameter, patch-level Bowen ratio (Eq. 13) is notable for TUF3D-calculated *Q*_*E*_ (e.g., a 27 % decrease for an increase of *β* from 0.1 to 0.3); therefore, there is likely greater uncertainty in *Q*_*E*_ compared to other fluxes predicted by TUF3D (less than 3.5 % change for Q*, *Q*_*H*_, and Δ*Q*_*S*_).

Based on source area *λ*_*V*_ of 15 % and linear regression coefficients in Grimmond and Oke (2002), mean *α* for this site is determined to be 0.29. By calculating *ΔQ*_*S*_ and *Q*_*E*_ with LUMPS (L2) using a range of *α* values, the greatest agreement between TUF3D- and L2-modeled *Q*_*E*_ occurs when *α* = 0.20. In the L2 model, this *α* value is equivalent to a *λ*_*V*_ of 1.6 %, so errors in land cover classification cannot explain this difference. To the extent that TUF3D is assumed accurate, this would suggest the empirical LUMPS *α-λ*_*V*_ relation is weighted towards more moist European and North American sites where vegetation is more likely to be irrigated and towards mid-latitude climates with different vegetation species with different hydrologic responses than this sub-tropical, arid, high-elevation location.

## Conclusions

Observations of Q* and *Q*_*H*_ from a lightweight low-rise neighborhood in an arid sub-tropical climate are presented and compared with a process-based urban climate model (TUF3D) and an empirical model (LUMPS). Observations also agree with results from measurements in a similar neighborhood-climate combination in Ouagadougou, Burkina Faso.

TUF3D Q* agrees well with measurements, and modest overestimation of *Q*_*H*_ may relate to horizontal advection or complexity unrepresented in the model. LUMPS *Q*_*H*_ agrees with measurements during the day, but underestimates *Q*_*H*_ at night. Overnight, this difference is enough to alter the sign of *Q*_*H*_ and thus is critical to applications that depend on atmospheric stability, such as modeling air pollutant mixing and dispersion.

Latent heat flux (*Q*_*E*_) is modeled using TUF3D and two implementations of LUMPS (both a forward application, and an inverse application where measured Q* and *Q*_*H*_ are used to partition the remaining energy between *Q*_*E*_ and Δ*Q*_*S*_). All three models indicate *Q*_*E*_ is a minor component of the energy balance at this site (1000–1500 LST Bowen ratio = 2.45–3.46). *Q*_*E*_ from both LUMPS versions is somewhat higher than TUF3D (by 13.0 W m^−2^ during daytime) This possibly reflects bias towards mid-latitude neighborhoods and non-arid climates in the derivation of the empirical LUMPS parameters.

The storage heat flux (*ΔQ*_*S*_) is modeled using OHM, the inverse application of LUMPS, and TUF3D. The three models demonstrate reasonable agreement, though there are notable differences. The diurnal course from TUF3D and inverse LUMPS show peak *ΔQ*_*S*_ preceding peak Q* by ~2 h, which also matches results from Ouagadougou, Burkina Faso (Offerle et al. 2005). In contrast, peak *ΔQ*_*S*_ from OHM occurs at the same time as peak Q*. This difference probably relates to a lack of OHM coefficients in the literature that describe the built characteristics at this site; in particular thin, un-insulated metal sheet roofs, and concrete block walls (vs. the choice of the closest analog used here). New OHM coefficients have been determined for the roofs and walls in this neighborhood (and for the entire neighborhood) based on modeled *ΔQ*_*S*_ and observed local-scale Q*. Measurements are required to test these new coefficients.

More measurements of energy balances in lightweight low-rise neighborhoods are required to improve empirical and process-based models. Of particular interest would be measurements of the hysteresis between net radiation and heat storage of metal roofing material and full energy-balance measurements in a broader range of neighborhoods and climatic conditions, especially those typically found in the Global South.
